# Mesenchymal stem cells avoid allogeneic rejection

**DOI:** 10.1186/1476-9255-2-8

**Published:** 2005-07-26

**Authors:** Jennifer M Ryan, Frank P Barry, J Mary Murphy, Bernard P Mahon

**Affiliations:** 1 Institute of Immunology, National University of Ireland, Maynooth, Co. Kildare Ireland; 2 Regenerative Medicine Institute (REMEDI), National Centre for Biomedical Engineering Science, National University of Ireland, Galway, Ireland

## Abstract

Adult bone marrow derived mesenchymal stem cells offer the potential to open a new frontier in medicine. Regenerative medicine aims to replace effete cells in a broad range of conditions associated with damaged cartilage, bone, muscle, tendon and ligament. However the normal process of immune rejection of mismatched allogeneic tissue would appear to prevent the realisation of such ambitions. In fact mesenchymal stem cells avoid allogeneic rejection in humans and in animal models. These finding are supported by in vitro co-culture studies. Three broad mechanisms contribute to this effect. Firstly, mesenchymal stem cells are hypoimmunogenic, often lacking MHC-II and costimulatory molecule expression. Secondly, these stem cells prevent T cell responses indirectly through modulation of dendritic cells and directly by disrupting NK as well as CD8+ and CD4+ T cell function. Thirdly, mesenchymal stem cells induce a suppressive local microenvironment through the production of prostaglandins and interleukin-10 as well as by the expression of indoleamine 2,3,-dioxygenase, which depletes the local milieu of tryptophan. Comparison is made to maternal tolerance of the fetal allograft, and contrasted with the immune evasion mechanisms of tumor cells. Mesenchymal stem cells are a highly regulated self-renewing population of cells with potent mechanisms to avoid allogeneic rejection.

## Review

### Introduction: What are Stem Cells?

**Figure 1:**
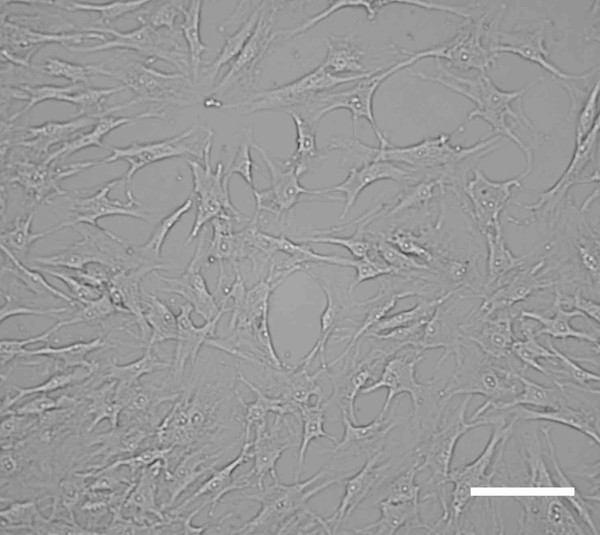
Human mesenchymal stem cells (MSC) are spindle shaped, fibroblast-like cells. Original magnification × 100, phase-contrast light microscopy, scale bar represents 50 μm.

The term "stem cell" can be applied to a remarkably diverse group of cells. These cells, regardless of their source, share two characteristic properties. Firstly, they have the capacity for prolonged or unlimited self-renewal under controlled conditions, and secondly they retain the potential to differentiate into a variety of more specialized cell types [1,2]. The stem cells that arise during the first days of mammalian embryonic development are pluripotent and are referred to as embryonic stem (ES) cells. These are usually derived from the inner cell mass of the pre-implantation embryo, at the blastocyst stage[3]. However stem cells are not confined to tissues of early development, but can also be found at various sites in the adult mammal. Adult stem cells are more differentiated then ES cells but can still give rise to specialized lineages[1,2]. The best-described populations to date are the hematopoietic stem cells (HSC) of the bone marrow that can generate various blood cells[4]. However the bone marrow also contains a population of mesenchymal stem cells (MSC) [1,2]. These cells, first characterized by Friedenstein and colleagues more than thirty years ago, are multipotent cells capable of differentiating into several lineages including; cartilage, bone, muscle, tendon, ligament and adipose tissue[2,5,6]. In their undifferentiated state, MSC are spindle-shaped and resemble fibroblasts[5,6] (Fig 1). There are no cell surface markers that specifically and uniquely identify MSC, and their characterization in the literature lacks consistency. The diversity of characteristics associated with MSC can be explained by differences in tissue origin, isolation methods and culture conditions between laboratories, in addition there appear to be strain-to-strain differences in murine derived MSC[2,7-9]. Whilst there is an obvious need for standardization between research groups, some consensus can be found among the conflicting data. In broad terms, MSC expanded in vitro do not express the hematopoietic or endothelial surface markers CD11b, CD14, CD31, CD34 or CD45 but stain positive for CD29, CD44, CD73, CD105, CD106 and CD166 [2,5,10]. The non-embryonic source of this population, the reduced likelihood of neoplasia, and the more limited differentiation potential, have made these cells attractive candidates for application in cell based therapies usually termed "regenerative medicine"[2]. There is one confounding influence on this approach; whilst self derived MSC pose few immunological problems, in practice regenerative medicine is likely to rely on mismatched (allogeneic) cells to repair or replace damaged tissue. Normally, allogeneic cells are deleted by host immune responses. The major surprise to Immunologists working in this field have been findings that suggest that MSC do not obey the normal "rules" of allogeneic rejection. This review will survey recent data, which convincingly indicate the mechanisms by which MSC escape the normal process of alloantigen recognition.

### MSC evade allorejection

The major limit to solid organ graft survival is T cell recognition by the recipient of alloantigen (dominated by, but not confined to MHC/HLA antigens)[11]. There are two mechanisms mediating this powerful rejection response; "direct" recognition, involving recognition by recipient CD8+ or CD4+ T cells of donor MHC class I and class II molecules; and "indirect" mechanisms involving recognition of peptides from the allogeneic tissue[11]. Recipient antigen presenting cells (APC) such as dendritic cells (DC) process alloantigen into peptides and present these to naive T cells on self-MHC molecules [12]. However there are notable exceptions to these allorejection processes; the fetal allograft evades rejection by the mother through a complex series of actions (reviewed in[13]), similarly tissue which has limited lymphatic drainage is less prone to allorejection[14]. Interestingly tumor cells, whilst not allogeneic, are in many cases both "altered-self" and immunogenic but often actively modulate immune responsiveness to evade immune surveillance[15]. Thus mechanisms of tumor evasion of the immune system may provide insight into how allogeneic MSC are tolerated by the mismatched host.

There is supporting evidence for the use of allogeneic MSC from both in vitro and in vivo studies that show MSC avoid normal alloresponses. A small number of in- vivo studies suggest that MSC play a role in enabling alloantigen tolerance. Koc et al, showed no evidence of alloreactive T cells and no incidence of graft v host disease when allogeneic MSC were infused into patients with Hurler's syndrome or metachromatic leukodystrophy[16]. In a previous study by the same group, autologous culture-expanded MSC were infused to breast cancer patients to investigate whether MSC would enhance the engraftment of peripheral blood stem cells after myeloablative therapy [17]. Results showed rapid hematopoietic recovery and no signs of toxicity from MSC infusion[17]. Horwitz and colleagues, reported that donor MSC contributed to bone remodelling after allogeneic stem cell transplantation in three children with osteogenesis imperfecta (OI)[18], a rare genetic disorder of type I collagen. This is supported by data from Bartholomew et al who showed that in-vivo administration of allogeneic MSC prolonged 3rd party skin graft survival in animal models[19]. Furthermore, Saito et al, demonstrated that MSC undergoing differentiation to a cardiac phenotype were tolerated in a xenogeneic environment, retaining their ability to be recruited to the injured myocardium[20]. More recent work by Aggarwal and Pittenger supported the feasibility of MSC-transplantation showing that MSC altered the phenotypes of specific immune cell subtypes thereby creating a tolerogenic environment[21]. These reports suggest that transplantation of MSC could be beneficial in patients with various disorders requiring tissue regeneration, and provide evidence supporting the tolerance of allogeneic MSC by recipients.

Data supporting the contention that MSC avoid allogeneic responses has also come from a large body of in vitro experiments, usually involving co-culture or mixed lymphocyte reactions (MLR). Evidence from these studies indicate that the use of mismatched MSC does not provoke a proliferative T cell response in allogeneic MLR, thus suggesting an immunosuppressive role for MSC[19,22-26]. Le Blanc et al, showed that MSC failed to elicit proliferation of allogeneic lymphocytes[27]. Additionally, they demonstrated that MSC remained immunosuppressive even after IFN-γ stimulation[27]. Evidence from Krampera et al confirms these findings, they showed that murine MSC lack MHC class II and inhibited T cell proliferation[25]. Tse et al, also showed that human MSC fail to elicit allogeneic T cell response in a MLR even when MHC class II was upregulated[28]. Consistent with these studies, Bartholomew et al showed that allogeneic baboon MSC suppressed the proliferative activity of lymphocytes in vitro and prolonged graft survival[19]. These findings support the view that MSC can be transplanted between MHC-incompatible individuals. Although these data show that successful use of allogeneic MSC in regenerative therapy is possible, such approaches are unlikely to be broadly acceptable until it is understood why MSC are not rejected. This question has been the subject of intense recent study and three candidate mechanisms are emerging. MSC appear to evade allogeneic rejection by a) being hypoimmunogenic; b) modulating T cell phenotype and c) creating an immunosuppressive local milieu. These mechanisms are inter-related and will involve cell contact dependent and independent interactions. The challenge facing the field is to unravel the contribution of these diverse interactions.

### MSC are hypoimmunogenic

**Figure 2:**
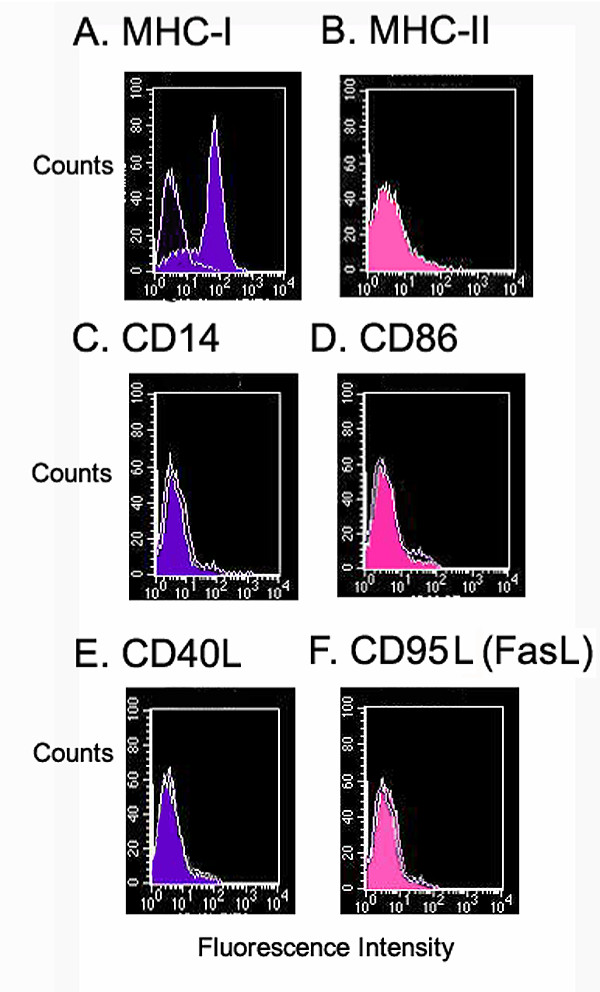
Human MSC cultured according to [106, 107] are A) MHC-I positive (HLA-A,B,C, antibody W6/32-FITC), B) MHC class II negative (HLA-DR, antibody LN-3-PE); C) CD14 negative (antibody MEM-18-FITC), D) CD86 negative (antibody IT2.2-PE); and E) CD40L/ CD154 (antibody 24-31-FITC), F) CD95L (FasL) negative (antibody NOK-1-PE). Isotype matched control antibody labelling are shown as unshaded plots, FITC conjugates are shown in blue, PE conjugates shown in pink. Flow cytometry performed according to methods previously described [108-110].

There is controversy surrounding the cell surface expression of MHC alloantigens by MSC. Although conflicting evidence exists, most studies describe human MSC as MHC class I positive and MHC class II negative (Fig 2). The data conflicting with these findings may represent different stem cell lineages or be the result of the recently described process of cell-cell transfer [29-31]. The expression of MHC class I by MSC is important because expression protects MSC from certain NK cell mechanisms of deletion. For instance, a major function of NK and NK-like cells is to kill tumor cells that have downregulated class I [32]. HLA-G is an MHC-like protein that is known to protect the fetal allograft against NK mediated rejection[33,34]. This protein has been shown to bind to the two major inhibitory NK receptors, KIR1 and KIR2, and to inhibit NK killing [35-37]. However no studies of HLA-G expression by MSC have been reported to date.

As MHC class II proteins are potent alloantigens, the expression by MSC is another important factor. Again there is some controversy over expression, which may be explained by the diversity of models described above. However there are widespread observations that under non-inflammatory conditions, human MSC are MHC-II negative, supporting a role for MSC as having reduced immunogenicity through the control of alloantigen expression [38-40]. The absence of MHC class II gives MSC the potential to escape recognition by alloreactive CD4+ T cells. In addition to being MHC II negative, MSC do not appear to express the co-stimulatory molecules CD40, CD40L, CD80 or CD86 required for effector T cell induction[28,39]. The absence of co-stimulatory molecules is a significant observation. It implies that any residual engagement of the T cell receptor on Th cells would result in anergy and contribute to tolerance rather than allogeneic responses. Although this is a comforting scenario, based largely on in vitro studies, it cannot fully explain the evasion of alloreactivity demonstrated by MSC. Experiments involving allogeneic co-cultures or MLR have demonstrated that both cell-cell contact and action by soluble factors contribute to the immunomodulatory function of MSC[25,41-43]. Thus it is likely that evasion of alloreactivity is a result of both MSC hypoimmunogenicity, modulation of T cell immune induction and the creation of a suppressive milieu around MSC. Although the mechanisms governing the suppressive effect are not fully understood, several studies have given indicators to the processes involved.

### MSC interfere with DC maturation and function

Dendritic cells (DC) are the most influential APC, playing a key role in directing cellular and humoral immune responses against self and non-self antigens [44]. DC contribute to the establishment of tolerance, especially in the periphery[45]. Immature DC are not fully differentiated to carry out their known roles as inducers of immunity[45]. Despite this, immature DC circulate through tissues and the lymph system, capturing self and non-self antigens[45]. Immature DC that are loaded with antigen can silence T cells by deletion or by expanding regulatory T cell populations[45,46]. It has long been believed that this process contributes to graft survival during transplantation [14]. The capacity of DC to induce peripheral tolerance is a potential mechanism by which MSC could manipulate immunity in order to escape T cell recognition. Thus MSC could prevent normal allogeneic responses either through modulation of DC function or by direct effects on T cells. Indications from different studies encourage this hypothesis. Zhang et al [24] provides evidence that MSC interfere with DC maturation. Co-culture experiments showed that MSC down-regulate CD1a, CD40, CD80, CD86, and HLA-DR expression during DC maturation[24]. This is also shown by Beyth et al. [42], who suggest that human MSC converted APC into an inhibitory or suppressor phenotype via cell-to-cell contact, thus locking DC into a semi-mature state and thereby inducing peripheral tolerance. Their findings also show reduced IFN-γ, IL-12 and TNF-α in human MSC/monocyte co-culture [42]. Similarly Jiang et al reported that MSC maintain DC in an immature state[26] and show that MSC inhibit up regulation of IL-12p70 [26]. These results suggest that MSC mediate allogeneic tolerance by directing APC towards a suppressor or inhibitory phenotype that results in an attenuated or regulatory T cell response.

### MSC modulate CD4+ T cell responses

Evidence has emerged that MSC interact directly with T cells to suppress alloreativity[25]. Krampera et al showed that MSC impair T cell contact with APC in a non-cognate but transient fashion[25]. This supported work from Bartholomew et al showing that the addition of IL-2 to MLR/MSC co-cultures reduced MSC suppression and restored T cell proliferation[19]. Taken together, these results strongly support a role for either a direct (T cell phenotype) or indirect (DC phenotype) mechanism of immune modulation directed by MSC.

MSC modulation of CD4+ T cell responses is more extensive than the straightforward effect described above. The regular process of antigen specific CD4+ T cell induction requires antigen capture and processing by DC (or other amenable cells), followed by a process of maturation and trafficking to local lymph nodes[14,47-49]. There is evidence that MSC prevent normal allogeneic responses by directing CD4+ T cells to a suppressive or counter-regulatory phenotype[46,50]. Di Nicola et al, showed that MSC strongly suppressed CD4+ (and CD8+) T cells in MLR[43], findings supported by Tse et al, who showed that MSC suppress the proliferation of T-cell subsets[28]. Studies of T cell differentiation have shown that in the presence of human MSC, Th1 cell secretion of IFN-γ dropped by 50% compared to cultures without MSC. Conversely, effector T cells undergoing Th2 differentiation when co-cultured with human MSC showed a significant increase in IL-4 production compared to controls[21]. These findings suggest that MSC exert a counter regulatory, anti-inflammatory role by directing cytokine-mediated immunity[21].

A strategy of regulation and deletion of specific T cells is an effective control against unwanted immune responsiveness especially after transplantion[51]. Consequently, enormous interest has focused on the possibility of Treg cells as a marker for T cell tolerance during transplantation. Treg can act directly on other T cells or indirectly through APC[46]. Aggrawal et al, demonstrated that CD4+ CD25+ T reg populations increased significantly in MLR when MSC were present compared to controls[21]. However, data exists showing that human MSC-mediated inhibition is not suppressed by removing T reg cells from co-cultures [25,42]. Nevertheless a role for these cells can not be excluded, it is possible that an incomplete replication of the suppressive microenvironment in vitro or indeed the diversity of Treg cell populations mean that these studies do not fully explore the potential role of suppressive or regulatory T cells in promoting MSC tolerance.

MSC influence control over cell division cycle pathways in cells of immunological relevance. Glennie et al have shown that T cells stimulated in co-cultures with MSC exhibit an extensive inhibition of cyclin D2 and upregulation of the cyclin dependent kinase inhibitor p27^kip1 ^[52]. As T cell inhibition could not be reversed, these cells were not interpreted as anergic in the classical sense. The authors suggest that MSC are most likely inducing the alternative condition of divisional arrest anergy in T cells, an occurrence usually associated with CTLA-4 signalling[53]. In addition, removal of MSC from the system only restored IFN-γ production but not T cell proliferation[52]. This suggests that MSC induce a condition similar to split anergy[54] or split tolerance[55,56]. The key point is that this work demonstrates that MSC exert veto effects on T cells and it is significant in demonstrating that the mechanisms inducing MSC tolerance are not confined to patterns of cytokine secretion but extend to direct modulation of T cell division.

### MSC modulate CD8+ T cell and NK cell activity

The impact of MSC on CD8+ CTL and NK cells has also been addressed. CTL can lyse allogeneic cells after recognition of cognate alloantigen, by the release of cytotoxic effectors such as, perforins, serine esterases, IFN-γ and TNF-α [57] whereas NK cells do not require antigen processing[58]. Consequently both effector cells can operate in tandem, with NK cells providing a first line defence killing target cells that escape CTL recognition or show inadequate expression of self-MHC[58]. There is evidence that MSC inhibit the formation of CTL and appear to evade NK cell targeting mechanisms. Djouad et al showed that CD8+ cells are suppressed by MSC in MLR[41]. Rasmusson supported these findings and further showed that NK cells in co-culture did not recognize MSC although lytic capability was still present[59]. This effect appeared to be mediated by soluble factors[50,59]. Thus MSC interact and suppress cell-mediated immune responses directly and through soluble factors. The targets for this suppression are DC, CD4+ Th, CD8+ CTL and NK cells; in effect MSC silence each aspect of the cellular rejection process.

### MSC secrete soluble factors to create an immunosuppressive milieu

The characterisation of cytokines produced by MSC is still provisional and is hindered by the lack of standardisation in isolation and culture conditions, which have given rise to multiple findings and interpretations. It is evident that MSC do not constitutively express IL-2, IL-3, IL-4 and IL-5[60,61]. However some reports show that MSC do constitutively express mRNA for cytokines such as interleukin (IL)-6, -7, -8, -11, -12, -14, -15, -27, leukaemia inhibitory factor, macrophage colony-stimulating factor, and stem cell factor[62,63]. Some of these cytokines provide critical cell-cell interactions and promote HSC differentiation, however caution should be exercised before over interpreting these findings. Protein secretion does not always mirror mRNA levels and most workers in the field would adopt a more conservative profile of cytokine and growth factor production by MSC.

Despite these caveats, certain MSC secreted products such as Hepatocyte growth factor, (HGF) are likely to contribute to creating a local immunosuppressive environment. HGF induces mitogenic and antiapoptotic activity in different systems [64-66] and has a well-characterized role in wound repair [66-68], effects that are consistent with a role for MSC in regenerative medicine. Although some groups do not detect HGF in MSC co-cultures [41] more reports suggest that HGF is constitutively expressed by MSC [13,43,69,70]. Indications that MSC produce HGF [13,43,69,70] encourage a role for these cells in tissue repair [70]. Studies by Chunmeng et al, demonstrated that rat dermal derived "multipotent" cells secrete HGF and promote wound healing[68]. Interestingly, Azuma et al, showed that HGF treatment prevents chronic allograft nephropathy in rats[71]. Taken together these results suggest that HGF may contribute to the ability of MSC to avoid allorejection.

IL-10 has a well-documented role in T cell regulation and in the promotion of a "regulatory" or suppressor phenotype. In our hands human MSC constitutively produce IL-10 whereas Rasmusson et al and Beyth et al only detected IL-10 in co-culture experiments [42,72]. In either case, IL-10 is likely to be suppressing potential allo-responsiveness because it is a recognized growth factor for regulatory T cells [73]. IL-10 can antagonize IL-12 during induction of inflammatory immune responses [74-79]. This is supported by studies showing that MSC partially mediate suppression through IL-10 secretion in MLR cultures[42,72]. Similarly transforming growth factor (TGF)-β1 also plays a role in T cell suppression. This cytokine as well as IL-10 influences cell lineages broader than lymphocytes [74,80,81]. However constitutive expression of TGF-β1 has not been detected from our own studies on human MSC[13]. This is in line with Le Blanc who found no difference in TGF-β1 concentration in co-cultures with or without MSC [69]. In contrast Beyth et al showed that TGF-β1 was secreted in media from co-cultures of human MSC and immune cells but again co-culture did not augment TGF-β1 concentration[42]. Although a number of studies suggest no role for TGF-β1 in evasion of allogeneic responsiveness[42,69,72], it has been suggested that HGF in combination with TGF-β promotes the allo-escaping phenotype[43]. Di Nicola et al showed that neutralizing antibodies to HGF and TGF-β restored the proliferative response in MLR, suggesting that these factors are at least partially responsible[43].

MSC constitutively express the eicosanoid Prostaglandin E (PGE)-2 [82]. This may be upregulated in co-culture[21,28] or downregulated on differentiation[82]. PGE-2 influences numerous immune functions including suppression of B cell activation[83] and induction of regulatory T cells[84]. Although there is evidence for PGE-2 secretion by MSC, there is controversy surrounding a role for PGE-2 as a mediator for suppression of alloresponses in MLR. Studies from Tse, suggested that PGE-2 is not a significant component of suppression[28]. Supporting these findings Rasmusson et al showed that blocking PGE-2 production did not restore allogeneic MLR responses but did influence mitogen driven proliferation[72]. Although the present opinions are conflicting, it should be highlighted that other possible prostaglandins and eicosanoids could be influencing alloresponses[85]. Analysis of these other immunomodulatory molecules could provide further clues as to how MSC escape the immune system.

In contrast to immunosuppression through the secretion of soluble factors, suppression may be mediated by withdrawal of factors in the micro-environment necessary for active immune responses. Indoleamine 2,3-dioxygenase (IDO) is an enzyme that catabolizes L-Tryptophan, thereby depleting an essential amino acid from the local environment [86-89]. Recent evidence has shown that this mechanism is exploited by the mammalian fetal allograft to suppresses T cell activity and prevent rejection [86-89]. Although not a soluble factor, the expression of IDO may contribute to a tolergenic environment. This is of great relevance and has obvious parallels with MSC. Meisel et al showed that IDO is not constitutively expressed by MSC but can be induced by IFN-γ[90], thereby inhibiting allogeneic T cell responses by Tryptophan depletion[90]. Other findings have suggested that IDO-mediated tryptophan depletion inhibits allogeneic T-cell responses by multiple pathways[91]. The discovery of this mechanism, which shows parallels to the creation of a "Tryptophan desert" at the materno-fetal interface[13], provides a further feasible mechanism by which MSC avoid alloreactivity. However, IDO expression is not essential to the maintenance of tolerance against MSC. Tse et al showed that an IDO inhibitor or supplementary Tryptophan addition to MLR did not restore PBMC proliferation [28].

MSC control surface marker expression to exhibit a hypoimmunogenic or tolerogenic phenotype. MSC can also modulate T cell induction directly or via DC and secrete a battery of immunosuppressive factors. It is apparent that the question facing the application of regenerative medicine is no longer "how do MSC escape alloreactivity?" but rather "what is the hierarchy of signals that control immunosuppression?" In this regard, research from other fields has been informative. We have previously proposed that maternal acceptance of the fetal allograft provides indicators of how this process is controlled[13]. However, insight could also come from another avenue of inquiry. The mechanisms of tumor evasion may reflect the survival mechanisms of MSC.

### MSC avoidance of alloreactivity shows parallels to tumor evasion

**Figure 3:**
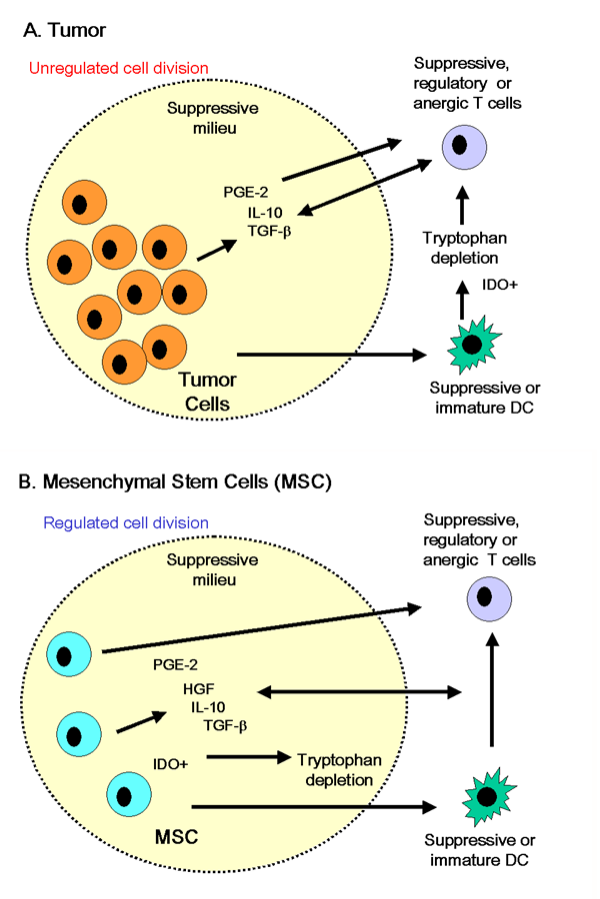
MSC and tumor cells create a suppressive microenvironment. There are fundamental differences between tumor cells (A) and MSC (B) with respect to control of cell division, however many mechanisms exploited by the former to evade immune deletion are also used by MSC to avoid allogeneic rejection. Details of mechanisms and associated references are supplied in the body of the text and Table 1.

Escape from immune surveillance is believed to be a primary feature of malignant disease in humans. The immune effector response is sub-optimal because tumors develop multifactorial strategies to escape immune deletion[92,93]. These strategies may provide clues to how MSC promote tolerogenic mechanisms during allogeneic engraftment (Fig. 3). Modulation of tumor antigen expression, particularly MHC class I and II is a particularly common component of tumor immune evasion[93]. This is often accompanied by poor or non-expression of co-stimulatory molecules, which not only limits clonal expansion of tumor-specific CD4+ T cells, but also hinders the production of cytokines, and the development of CTL[44,94,95]. Similarly MSC show no expression of co-stimulatory molecules (Fig. 2) [28,39]. In addition to reduced immunogenicity, tumor cells can directly modulate DC and T cell function. Studies from patients with hepatocellular carcinoma showed that neoplasia induced a defect of DC maturation[96]. This parallels findings by Beyth et al [42] suggesting that human MSCs interfere with normal APC maturation, thereby indirectly influencing T-cell activation. Freshly isolated tumor-infiltrating T cells are usually inactive against autologous cancer cells but can be reactivated in-vitro by the addition of IL-2[97]. Studies of MSC by Le Blanc et al showed striking parallels to this form of suppression[69]. They suggest that MSC act by preventing expression of CD25 (IL-2 receptor) thereby limiting T cell activation[69]. Other work has shown that exogenous IL-2 addition to co-cultures containing MSC reversed the suppressive effect[19,69]. Similarly, antigen-specific CD4+ CD25+ regulatory T cells also suppress tumor-specific CD8 T cell cytotoxicity although this mechanism relies on TGF-β secretion by regulatory cells[98,99].

Tumors can suppress CD4+ T cell activity and CTL tumor lysis directly through secretion of immunosuppressive factors including TGF-β1 but also PGE-2, and IL-10. Van der Pouw Kraan et al, showed that tumor-derived prostaglandins increased the production of inhibitory cytokines such as IL-10, while suppressing IL-12[100], which is necessary for effective host-cell-mediated anti-tumor immune response[75,93]. Likewise, TGF-β production has been reported from a number of tumors, contributing to immune evasion. Intriguingly in this context it also inhibits CTL differentiation [101]. Although there is little evidence that MSC secrete TGF-β1, the bone marrow is rich in this cytokine, suggesting that MSC reside in a compartment with immunosuppressive qualities.

Although there are striking parallels between MSC and some tumor cells, it is not our contention that these cells are directly related. Indeed there are distinct differences between the populations (Table 1). The fundamental difference between the cell types resides in the control of cell division and apoptosis, which are tightly regulated in MSC but dysregulated in transformed cells. Furthermore, it is well documented that some tumors exploit FasL (CD95L) expression to facilitate immune escape [102-104]. However, our own studies show that human MSC do not express FasL (Fig 2) and although there is some evidence from immortalized mini-pig derived MSC to indicate a role for FasL in suppression[105], it seems that direct induction of apoptotic deletion is not a factor involved in MSC interaction with T cells in the broader literature. The parallels between neoplastic cells and MSC lie in the expressed phenotypes rather than in any direct lineage relation. It appears that MSC retain certain aspects of the fetal allograft that promote tolerance, some of these mechanisms may be reactivated in neoplasia, the key difference being that MSC perform these functions in an ordered and controlled way whereas tumor cells do so in a manner that by definition has escaped normal controls on apoptosis or cell division.

**Table 1:** Comparison of MSC and Tumor cells^a^

**Characteristic**	**MSC**	**Tumor cells**	**References**
Cell Division	Controlled	Uncontrolled	[5, 7, 111]
MHC I expression	+	Variable	Fig 2 & [25, 27, 28, 39, 93, 111, 112]
MHC II expression	-	Variable	Fig 2 & [2, 25, 27, 39, 93, 111, 112]
CD80 expression	-	-	[25, 28, 39, 44, 94, 95]
CD86 expression	-	-	Fig 2 & [25, 28, 39, 44, 94, 95]
FasL expression	-	+	Fig. 2 & [102-104]
Prostaglandin secretion	+	+	[21, 28, 82, 100]
IDO expression	+	Variable	[28, 43, 59, 87, 90]
TGF-β secretion	Variable	+	[42, 43, 59, 101, 105]
IL-10 secretion	+	+	[13, 42, 72, 100]
DC modulation	+	+	[24, 26, 42, 96]
Veto effects on T cells	+	+	[23, 112]

a Descriptions of MSC in the literature are diverse and many populations have been described which show different patterns of expression. In particular work in mice appears to be strain dependent, but further variation arises from differences in isolation, culture, timing and methodology. Likewise the characteristics of neoplastic cells will vary greatly between different tumors. This table lists those characteristics where at least some cells from each diverse population show either comparative or contrasting features.

## Conclusion

Current research on the interaction between MSC and T cells support the potential use of allogeneic MSC in regenerative medicine. Studies showing enhanced MSC engraftment of bone, muscle, heart etc encourage the translation of recent research into therapy. The future holds much promise for the use of allogeneic MSC and whilst obstacles exist, the potential for alloreactivity does not seem to be a major problem. From the research standpoint, MSC appear to use a surprising array of mechanisms to avoid deletion by the host including hypoimmunogenicity, modulation of DC and T cell function, as well as the creation of a suppressive microenvironment. The challenge is now to unravel the timing and control of these mechanisms in an inflammatory situation typical of the recipient patient.

## List of Abbreviations

APC, antigen presenting cells; DC, dendritic cell; ES, embryonic stem; HGF, hepatocyte growth factor; HSC, hematopoietic stem cells; IDO, indoleamine 2,3,dioxygenase; KIR, killer inhibitory receptor; MLR, mixed lymphocyte-like reaction; MSC, mesenchymal stem cells; OI, osteogenesis imperfecta; PBMC, peripheral blood mononuclear cells; PGE-2, prostaglandin E2.

## Competing interests

JMR and BPM have no competing interests. FPB and JMM have received salary from an organization and hold stocks or shares in an organization that may gain or lose financially from the publication of this manuscript.

## Authors' contributions

FPB and BPM conceived the review; JMR performed the microscopy and flow cytometry. All authors provided interpretation of published stem cell data, and have made intellectual contributions to the content of the paper. All authors read and approved the final manuscript.

## Supplementary Material

Additional File 1 Ryan et al library. Click here for file
